# Case Report: Management of cervical intramedullary spinal cord metastasis from NSCLC with a literature review

**DOI:** 10.3389/fsurg.2026.1760091

**Published:** 2026-02-25

**Authors:** Hua Liu, Long Chen, Feng Li, Mingjiu Zhang, Tao Zhang, Songkai Li

**Affiliations:** 1 Department of Spinal Surgery, The 940th Hospital of the Joint Logistic Support Force of Chinese People’s Liberation Army, Lanzhou, Gansu, China; 2 First Clinical Medical School, Gansu University of Chinese Medicine, Lanzhou, Gansu, China; 3 Department of Orthopedics, The 943rd Hospital of the Joint Logistic Support Force of the Chinese People's Liberation Army, Wuwei, Gansu, China

**Keywords:** case report, cervical spine, intramedullary spinal cord metastases (ISCM), literature review, multimodal therapy, non-small cell lung cancer (NSCLC), survival

## Abstract

**Background:**

Intramedullary spinal cord metastases (ISCM) from non-small cell lung cancer (NSCLC) are rare and carry a grave prognosis. Cervical segment involvement is exceptionally uncommon, and its distinct clinicopathological profile is not well characterized.

**Methods:**

We present the case of a 72-year-old male with a history of NSCLC who developed acute quadriparesis and sphincter dysfunction. Cervical magnetic resonance imaging (MRI) revealed a C7 intramedullary mass. The patient underwent C6-T1 laminectomy with microsurgical gross-total resection. Histopathology confirmed metastatic lung adenocarcinoma. We supplemented this case with a systematic literature review of NSCLC-derived ISCM cases to summarize demographic, clinical, and therapeutic outcomes.

**Results:**

Histopathology confirmed metastatic lung adenocarcinoma. Postoperatively, the patient's neurological function improved. Although local recurrence was detected at 11 months and treated with salvage radiotherapy, the patient nevertheless maintained ambulatory function and was alive at the 18-month follow-up. Our literature review of 68 cases with complete data identified a male predominance (4.2:1 ratio) and a mean age of 58.1 years. The cervical spine was the most commonly involved segment (47.1%). Analysis of treatment modalities revealed that multimodal therapy, particularly the combination of surgery and chemotherapy (potentially incorporating modern agents such as immune checkpoint inhibitors), was associated with improved survival, with a mean overall survival of 15.0 months in this subgroup. This paradigm, centered around maximal safe resection, successfully achieved long-term functional preservation and survival.

**Conclusion:**

Cervical ISCM from NSCLC represents one of the most challenging complications in spinal oncology. This case, supported by our literature review, provides a surgical-led, multimodal management template for spine surgeons, demonstrating that aggressive yet strategic intervention can achieve favorable long-term neurological and survival outcomes.

## 1 Introduction

Intramedullary spinal cord metastases (ISCM) represent a rare and devastating complication of systemic cancer, occurring in only 0.1%–0.4% of all cancer patients and accounting for 1%–3% of all intramedullary spinal neoplasms (1). Importantly, ISCM constitutes an oncological emergency due to the risk of rapid and irreversible neurological damage. Furthermore, in a significant proportion of cases (approximately 20%), ISCM may be the initial presenting manifestation of an otherwise occult malignancy, underscoring the critical need for heightened clinical suspicion and prompt investigation in patients with acute myelopathy, even in the absence of a known cancer history (2). Distinct from primary intramedullary tumors, ISCM is characterized by rapid neurological deterioration, typically progressing within days to weeks, and has traditionally been associated with a dismal prognosis, with historical median survival of merely 3–6 months (3, 4). The management of spinal metastases constitutes a critical subspecialty within spine surgery, focusing on preserving neurological function and structural stability. Complementing these core objectives, a comprehensive multimodal strategy should also incorporate structured neurorehabilitation. Early involvement of rehabilitation services is crucial to address pain, optimize functional recovery, manage neurogenic bowel and bladder dysfunction, and ultimately improve the patient's quality of life, which is a central goal in the management of metastatic spinal disease (5). Among these, ISCM presents the ultimate surgical challenge due to their location within the core of the spinal cord conduit, necessitating not only advanced microsurgical techniques but also integrated multimodal strategies to optimize outcomes. Epidemiological analyses consistently identify lung cancer as the predominant etiology of ISCM, accounting for approximately 20% of all cases overall, followed by breast cancer, melanoma, renal cell carcinoma, and lymphoma (6). A retrospective single-center study further highlighted prognostic factors specific to lung cancer-related ISCM: among NSCLC patients, those with EGFR mutations, ambulatory status, and an ECOG performance status of 1–2 had improved overall survival, whereas the development of motor deficits within ≤10 days of symptom onset was a negative prognostic factor (7). While the clinical features of small cell lung cancer (SCLC)-related ISCM have been reasonably well-documented in the literature, non-small cell lung cancer (NSCLC)-derived ISCM—particularly those with isolated involvement of the cervical spine—remains profoundly rare and undercharacterized. This scarcity of data complicates timely diagnosis and hinders the development of optimized management strategies.

The distinctiveness of our case is the sustained survival exceeding 18 months, which was achieved through a proactive, multimodal therapeutic approach. This favorable outcome offers a counterpoint to the traditionally poor prognosis and provides a rationale for aggressive, multimodal management. Herein, we report this instructive case and integrate its findings with a contemporary literature review to elucidate the clinical characteristics, prognostic determinants, and evolving treatment paradigms for this aggressive disease. This report aims to present a contemporary, surgical-led management paradigm for cervical ISCM, integrating advanced microsurgical resection with modern systemic therapies, and to validate this approach through a comprehensive review of the literature.

## 2 Case presentation

A 72-year-old male with a three-decade heavy smoking history underwent right upper lobectomy with mediastinal lymphadenectomy (stations 7 and 10) for primary lung cancer two years before the current presentation. Histopathological analysis of surgical specimens confirmed stage T1N0M0 pulmonary invasive adenocarcinoma (non-small cell type). The patient completed four cycles of platinum-based adjuvant chemotherapy (nedaplatin 80 mg/m^2^ + pemetrexed 500 mg/m^2^) with subsequent monthly surveillance protocol including thoracic CT and serum tumor marker profiling. Serial carcinoembryonic antigen (CEA) monitoring demonstrated progressive biochemical remission (959.63 ng/ml to 57 ng/ml to 9.71 ng/ml to 4.77 ng/ml; reference range <5 ng/ml), correlating with radiologic absence of recurrence. Three months prior to admission, the patient reported spontaneous cervicothoracic pain [Visual Analog Scale (VAS) 5/10] with bilateral limb dysesthesia refractory to nonsteroidal anti-inflammatory drugs. Acute neurological deterioration occurred 24 h prior to admission, characterized by gait ataxia, plantar paresthesia (described as “walking on cotton”), and neurogenic bladder dysfunction presenting as dysuria.

## 3 Timeline of care

Table 1.

**Table 1:** Clinical timeline, management, and outcomes.

Time point	Key events and management pathway
Initial Diagnosis (T-24 months)	Event: Diagnosis of primary NSCLC. Finding: Chest CT showing right upper lobe mass. Intervention: Right upper lobectomy; Adjuvant Chemotherapy (nedaplatin/pemetrexed ×4). Outcome: Disease-free for 24 months.
Symptom Onset & Diagnosis (T-0)	Event: Acute pain, paresthesia, urinary retention. Finding: Neurological exam (ASIA D, JOA 10/17); Elevated CEA (14.36 ng/ml); Cervical MRI confirming C7 intramedullary mass with rim enhancement. Intervention: C6-T1 Laminectomy & Tumor Resection. Outcome: Histopathological confirmation of metastatic lung adenocarcinoma.
Adjuvant Phase (T-1 to 6 months)	Event: Recovery and systemic treatment. Intervention: Resumption of adjuvant chemotherapy. Outcome: Neurological improvement (JOA 12/17); significant pain reduction.
Recurrence & Management (T-11 months)	Event: Asymptomatic biochemical and radiological recurrence. Finding: Elevated CEA (5.23 ng/ml); MRI showing a 7 mm nodule at C7. Intervention: Salvage Radiotherapy (40 Gy/20 fx). Outcome: Disease stabilization.
Most Recent Follow-up (T-18 months)	Event: Stable disease. Finding: Serial imaging shows no progression. Outcome: Patient alive and ambulatory with a cane.

ASIA, American Spinal Injury Association; JOA, Japanese Orthopaedic Association; CEA, Carcinoembryonic Antigen.

## 4 Diagnostic assessment, therapeutic intervention, follow-up, and outcomes

### 4.1 Diagnostic assessment

**Figure 1:**
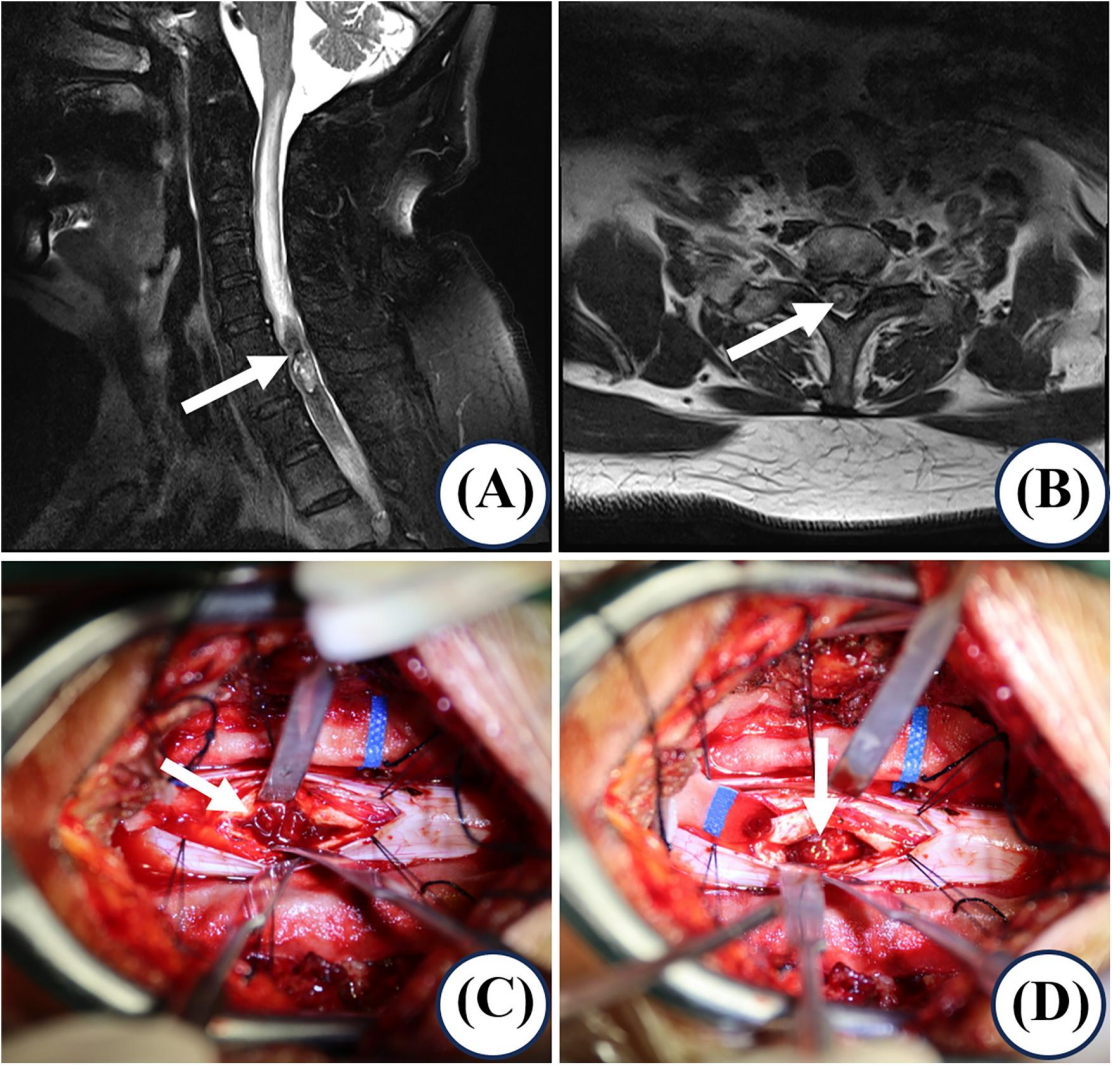
Diagnostic imaging and intraoperative confirmation. **(A,B)** Preoperative cervical MRI reveals a well-demarcated intramedullary lesion at the C7 level (2.0 × 1.5 × 0.8 cm, measured on radiologic sequences with mild spinal cord edema contributing to minor dimensional differences compared to intraoperative findings), demonstrating heterogeneous isointense to mildly hyperintense signals on T2-weighted imaging with homogeneous moderate enhancement. Characteristic imaging features include a peripheral “flame sign” (arrow in A) and a “central dot sign” on the axial section (arrow in B). **(C,D)** Intraoperative photographs identify an intradural tumor posterior to the C7 vertebral body, measuring approximately 1.6 cm × 0.8 cm × 0.8 cm (initial gross measurement prior to complete tumor exposure), with a relatively distinct boundary from the spinal cord, soft in texture, and grayish-white in appearance. The tumor was resected in a piecemeal fashion, achieving maximal tumor removal and decompression of the spinal cord.

Upon presentation with acute myelopathic symptoms, neurological examination demonstrated right lower extremity weakness (quadriceps strength grade 3/5), hyporeflexia, and positive upper motor neuron signs (Hoffmann's and Babinski's). The American Spinal Injury Association (ASIA) Impairment Scale was graded D, corroborated by a Japanese Orthopaedic Association (JOA) score of 10/17. A key diagnostic finding was the re-elevation of serum carcinoembryonic antigen (CEA) to 14.36 ng/ml. Subsequent contrast-enhanced cervical magnetic resonance imaging (MRI) identified a C7 intramedullary mass causing significant cord compression. The lesion exhibited heterogeneous enhancement, associated edema, and characteristic radiologic features highly suggestive of metastasis, including the “rim sign” and “flame sign” (Figures 1A,B). The primary diagnostic challenge was to differentiate this mass from a primary intramedullary tumor (e.g., ependymoma or astrocytoma), a distinction crucial for management that was ultimately confirmed by histopathological examination post-resection. For intracranial staging, a dedicated brain MRI was not performed prior to the emergency spinal surgery. However, the preoperative cervical MRI sequences, which included coverage of the posterior fossa and lower brainstem, showed no evidence of metastatic lesions or leptomeningeal enhancement in the visualized regions. In the postoperative period, a head CT scan was obtained, which also revealed no gross intracranial pathology. It is acknowledged that these findings do not constitute a complete contrast-enhanced brain MRI, which remains the gold standard for excluding small brain metastases and leptomeningeal disease.

### 4.2 Therapeutic intervention

**Figure 2:**
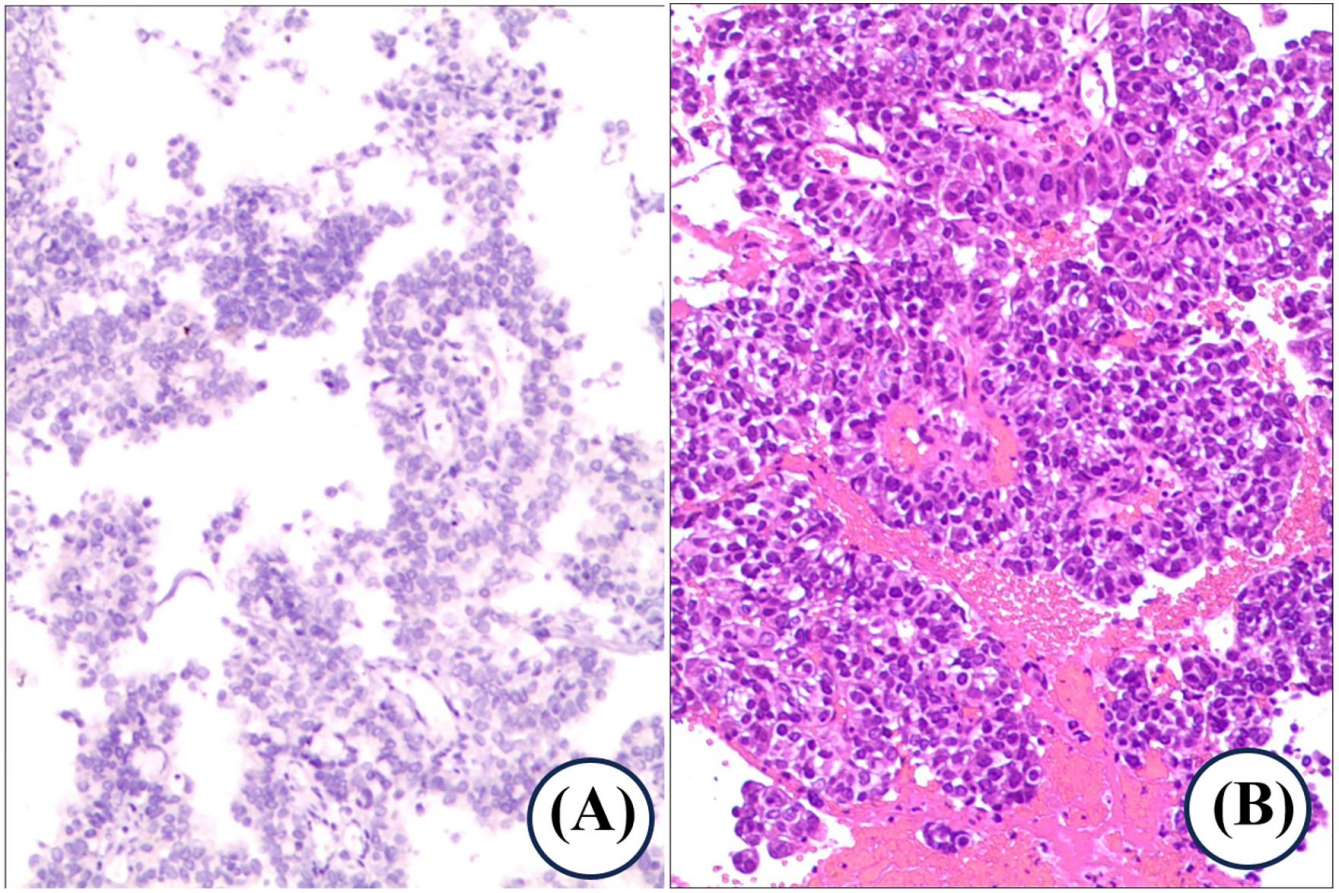
Histopathological confirmation of metastatic adenocarcinoma. **(A,B)** Histopathological examination of the resected specimen shows marked cellular pleomorphism, consistent with metastatic adenocarcinoma. Immunohistochemical (IHC) profiling demonstrated a diffusely positive immunoprofile with CK7, CK20, CKpan, TTF-1, and napsin-A expression, while S100, CgA, CD56, and GATA3 were negative. The Ki-67 proliferation index approximated 60%, and synaptophysin exhibited focal weak positivity. Molecular analysis identified wild-type EGFR status, and Ventana anti-ALK (D5F3) immunostaining was non-reactive. Histomorphological evaluation revealed marked cellular pleomorphism, consistent with metastatic adenocarcinoma. Combined IHC and molecular findings collectively supported the diagnosis of metastatic adenocarcinoma with probable pulmonary origin.

Upon radiographic confirmation of the intramedullary mass causing acute myelopathy, a multidisciplinary tumor board recommended emergency surgical intervention given the rapid neurological decline (within 24 h of symptom onset). The decision-to-incision interval was within 36 h of admission. Intraoperative neuroprotection was enhanced with a single intravenous dose of methylprednisolone (500 mg). Postoperatively, intravenous dexamethasone (10 mg daily) was administered for three days to manage spinal cord edema. The patient subsequently underwent a C6-T1 laminectomy with intradural tumor resection under general anesthesia. The entire procedure was conducted with continuous intraoperative neuromonitoring (IOM) of somatosensory and motor evoked potentials. After positioning, a midline posterior incision was made from C5 to T2 under C-arm fluoroscopic guidance for accurate level localization. The paraspinal muscles were dissected subperiosteally to expose the C6-T1 laminae. Laminectomy was performed *en bloc* using an ultrasonic bone scalpel and rongeurs. The dura was opened midline and retracted with stay sutures. Under the operating microscope, the spinal cord was incised longitudinally along the posterior median septum. A soft, grayish-brown intramedullary tumor measuring approximately 2.0 × 1.0 × 1.0 cm was identified at the C7 level. The tumor was meticulously resected in a piecemeal fashion using microdissectors and bipolar cautery, achieving gross-total resection. After tumor removal and hemostasis, the dura was closed watertightly with a 5-0 Prolene continuous suture and reinforced with a neurosurgical collagen matrix patch. The resected C6-T1 laminae and spinous processes were anatomically reduced and fixed *in situ* using titanium plates and screws. A closed suction drain was placed. The total operative time was 4 h and 50 min. Intraoperative IOM remained stable throughout the critical stages of resection (Figures 1C,D). Histopathological analysis confirmed metastatic adenocarcinoma. The pulmonary origin was verified by immunohistochemistry (positive for TTF-1 and CK7), while molecular profiling revealed no targetable mutations in EGFR or ALK (Figures 2A,B). Postoperatively, the patient's original systemic chemotherapy regimen of nedaplatin and pemetrexed was reinstated.

### 4.3 Follow-up, and outcomes

**Figure 3:**
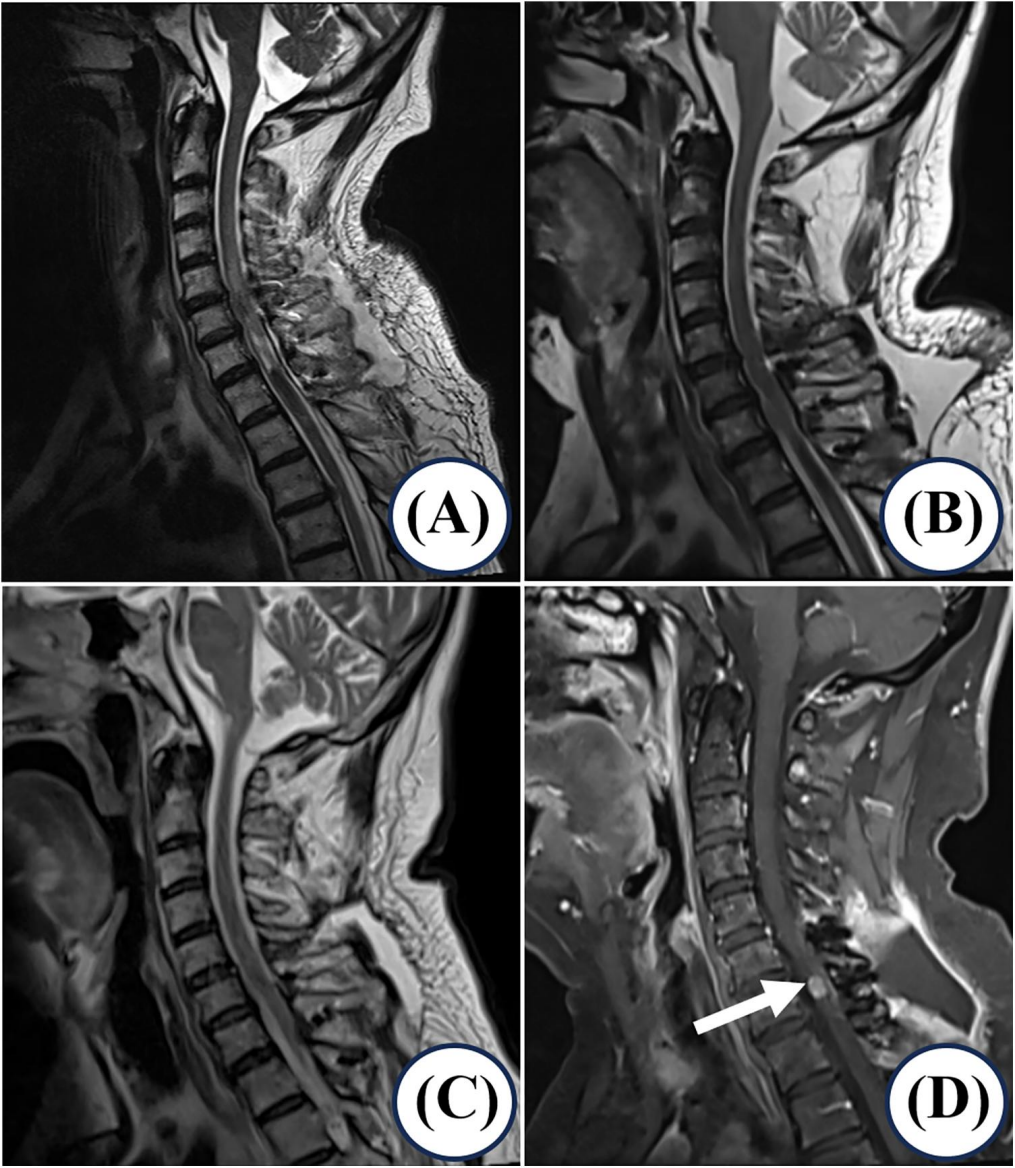
Postoperative imaging follow-up demonstrating treatment response and late recurrence. **(A)** Immediate postoperative MRI demonstrates expected postoperative changes, with a small posterior epidural fluid collection at the surgical site, consistent with a minor cerebrospinal fluid (CSF) leak. **(B)** The 3-month follow-up cervical MRI shows an interval increase in the volume of subcutaneous and epidural fluid along the posterior surgical tract from C5 to T1, indicating a persistent CSF leak. **(C)** A 6-month follow-up cervical MRI shows partial resolution of the previously noted subcutaneous edema and cystic formation. **(D)** At the 11-month follow-up, contrast-enhanced MRI identifies a 7-mm ring-enhancing nodular lesion (arrow) with T2-weighted fat-suppressed hyperintensity at the C7 level, resulting in mild spinal cord compression.

The patient's postoperative course was notable for significant functional improvement. A minor, asymptomatic cerebrospinal fluid (CSF) leak was identified at the surgical site on immediate postoperative imaging (Figure 3A) and was managed conservatively. Follow-up imaging demonstrated its natural evolution: the fluid collection increased in volume on the 3-month MRI (Figure 3B), likely related to postoperative changes and positioning, before showing significant—though incomplete—resolution by the 6-month follow-up (Figure 3C), consistent with gradual spontaneous healing. At the three-month follow-up, he reported reduced pain (VAS 3/10) and showed an improved JOA score of 12/17. Serial CEA levels remained within normal limits (2.1–4.8 ng/ml) for the first six months. However, at the 11-month evaluation, biochemical recurrence was detected (CEA 5.23 ng/ml), with corresponding MRI evidence of a new 7-mm nodular enhancement at the C7 surgical bed (Figure 3D). Salvage radiotherapy (40 Gy in 20 fractions) was administered to the recurrent lesion, which was well-tolerated, leading to disease stabilization. The patient achieved remarkable neurological recovery, regaining the ability to ambulate with a cane. At the most recent follow-up, 18 months after the initial spinal surgery, the patient remains alive with preserved neurological function, a survival duration that considerably exceeds the historical median for this condition. No major surgery- or radiotherapy-related complications were observed during the treatment and follow-up period.

## 5 Discussion

We present a case of cervical ISCM from NSCLC in a 72-year-old male who achieved sustained survival exceeding 18 months with multimodal therapy, challenging the traditionally dismal prognosis of this condition. By analyzing our management experience and reviewing contemporary literature, we summarize the characteristics of this disease to provide insights for clinical decision-making. The following discussion integrates our findings with the existing body of knowledge, highlighting strengths, limitations, and clinical implications.

### 5.1 Strengths and limitations

The principal strength of this report lies in the comprehensive documentation of the diagnostic and therapeutic journey, providing a viable and valuable management template for clinicians confronting this highly aggressive disease. The integration of a systematic literature review, analyzing 68 cases with complete demographic data, adds significant context and generalizability to our findings. However, the inherent limitations of a single-case design must be acknowledged. Our experience cannot establish standardized protocols, and the favorable outcome may be influenced by this specific patient's tumor biology, preoperative functional status (ASIA D), and the oligometastatic nature of his recurrence, factors not universally applicable. Meanwhile, the intracranial staging in this study relied on preoperative cervical spine MRI with limited coverage and postoperative head CT, rather than complete contrast-enhanced brain MRI, which may have impacted the comprehensive assessment of asymptomatic brain metastases.

### 5.2 Discussion in relation to relevant literature

Although lung cancer—the leading cause of cancer mortality (8)—is the most common ISCM origin, ISCM specifically arising from non-small cell lung cancer (NSCLC) is uncommon, with cervical involvement being exceptionally rare. Consequently, standardized diagnostic and therapeutic protocols for NSCLC-derived ISCM are lacking. We reviewed published NSCLC-derived ISCM cases (Supplementary Table S1) and compared them with our case to summarize key characteristics. Our literature review identified 77 reported cases of NSCLC-derived ISCM. Subsequent analyses of demographic and segment distribution were based on the 68 cases with complete data on age, gender, and lesion location to ensure consistency. Survival analyses were performed on the subset of patients with available survival time data.

#### 5.2.1 Clinical and demographic profile

Our analysis of 68 NSCLC-derived ISCM cases with complete demographic and topographic data reveals key characteristics of this disease. ISCM exhibits a significant male predominance (male-to-female ratio of 4.2:1) with a mean age of 58.1 years (1, 9), consistent with our 72-year-old male patient; notably, males face double the mortality risk of females (10), potentially attributable to underdiagnosis in females due to more insidious presentations (9). Regarding risk factors, nicotine promotes brain metastasis in lung cancer (11), likely contributing to our heavy-smoking patient's primary and metastatic disease, although a direct nicotine-ISCM link remains unproven. Clinically, manifestations include pain, numbness/sensory deficits, weakness/paraplegia, sphincter dysfunction, incontinence, and gait disturbances, which may rapidly progress to complete paraplegia; while ISCM resembles epidural metastases clinically, it shows a higher frequency of Brown-Séquard syndrome (3).

In terms of affected segments, our analysis of these 68 cases solidifies cervical involvement (47.1%, 32/68) as the most common site, aligning with reports of cervical vulnerability (1, 9), challenging prior reports of thoracic predominance (12–15), followed by thoracic (25%, *n* = 17), lumbar (5%, *n* = 4), conus medullaris (7%, *n* = 5), and multisegmental involvement (>3 segments, 14%, *n* = 10). This distribution corroborates Kalayci's observation of infrequent multisegmental involvement (1) and is consistent with our patient, who had a cervical lesion spanning fewer than three segments. Regarding prognosis, among 60 patients with survival data, mean survival was 10.79 months for males vs. 6.69 months for females, with 47 deaths and 13 survivors; this apparent male survival advantage may reflect limited historical female sample size and improved outcomes from modern microsurgery/multimodal therapy, consistent with Wu et al.'s report of better overall survival in females experiencing post-treatment symptom improvement (10).

For imaging, MRI is the cornerstone diagnostic tool (16, 17), with gadolinium-enhanced MRI demonstrating high sensitivity (1, 3, 18, 19). When ISCM is clinically suspected, MRI of the entire spine should ideally be obtained within 24 h to localize the lesion and assess for multifocal disease. Immediate initiation of corticosteroid therapy is standard to mitigate cord edema, followed by definitive treatment (e.g., surgery or radiotherapy), which should also be pursued urgently, ideally within 24 h of confirmation, to preserve neurological function (20). Characteristically, ISCM exhibits peripheral rim or nodular enhancement (“rim sign” and “flame sign”)—key distinguishing features from primary tumors (21, 22)—alongside T2 hyperintensity, intense enhancement, small size relative to primary tumors, normal cord caliber, and disproportionate peritumoral edema (23), all features present in our case (Figures 1A,B). Finally, differential diagnosis requires distinction from: primary intramedullary tumors (e.g., ependymoma), which often cause symmetric fusiform expansion with slow growth and may show a “cap sign” [19]; and intradural extramedullary tumors (e.g., meningioma), which typically present with radicular pain, cause cord displacement with homogeneous enhancement, and may exhibit a “dural tail sign” (23).

#### 5.2.2 Treatment modalities and survival outcomes

Current therapeutic approaches include radiotherapy, chemotherapy, surgical resection, palliative care, and multimodal therapy. The optimal sequencing of therapies must be individualized and is heavily influenced by tumor histology and biology. For example, in highly chemosensitive, systemic diseases such as primary spinal Ewing sarcoma, upfront chemotherapy may be a viable and effective strategy to achieve neurological improvement, challenging a uniform surgical-first approach (24). In contrast, for metastatic ISCM from solid tumors like NSCLC, where rapid cytoreduction and decompression of a well-demarcated lesion are often the goals, surgical resection—as demonstrated in our case—provides immediate and frequently irreplaceable benefits. Among reviewed cases and our report: 16 received radiotherapy alone, 5 surgery alone. Combination therapies included: surgery + radiotherapy (9 cases), surgery + chemotherapy (3 cases), radiotherapy + chemotherapy (16 cases). Ten patients underwent multimodal therapy (surgery + radiotherapy + chemotherapy). Mean survival was: surgery alone 2.5 months, radiotherapy alone 5.8 months; combination therapy: surgery + radiotherapy 7 months, surgery + chemotherapy 15 months, radiotherapy + chemotherapy 11.8 months; Most compellingly, multimodal therapy (surgery + radiotherapy + chemotherapy) demonstrated superior survival over single/dual-modality approaches, with a mean survival of 24.6 months. Our patient's management trajectory—initial surgery followed by chemotherapy—reflects this combinatorial strategy. At 11-month follow-up, mild CEA elevation (5.23 ng/ml) prompted supplemental radiotherapy (40 Gy/20 fractions), yielding symptom stabilization and pain reduction. Radiotherapy typically halts tumor growth and stabilizes neurological decline (3, 6) but poses tolerance and myelotoxicity concerns in elderly patients. It remains a safe option for radiosensitive ISCM (e.g., small cell carcinoma, lymphoma). Surgery may benefit patients with favorable functional status, limited systemic disease, and good prognosis (6, 15, 25).

Systemic therapy, particularly modern systemic agents, plays a crucial and expanding role in the management of ISCM. In recent years, cancer pharmacotherapy has diversified significantly, with survival benefits now achievable not only through conventional cytotoxic chemotherapeutic agents but also via molecularly targeted agents and immune checkpoint inhibitors. For the management of ISCM secondary to advanced NSCLC, cisplatin combined with pemetrexed represents the well-tolerated and conveniently administered first-line treatment (26). Additionally, molecularly targeted drugs offer three main options: agents targeting the epidermal growth factor receptor (EGFR) mutation (osimertinib, afatinib, gefitinib), which have demonstrated efficacy against NSCLC-associated ISCM (27–29); ALK inhibitors (e.g., ceritinib, lorlatinib) that readily penetrate the blood-brain barrier (30, 31); and immune checkpoint inhibitors targeting PD-L1. Notably, approximately 25%–36% of NSCLC tumors express PD-L1 (32), and nivolumab, which inhibits PD-L1, has been established for NSCLC treatment, representing a potential option for patients with asymptomatic small solitary ISCM (33, 34). In our reported case, favorable maintenance was achieved using sustained pemetrexed combined with nedaplatin, which exhibits lower nephrotoxicity than cisplatin.

#### 5.2.3 The pivotal role of spine surgery in ISCM management

Our experience, consistent with the literature, positions surgical intervention as a cornerstone in the modern, multimodal management of select patients with ISCM. The rationale for surgery is threefold and often irreplaceable by other modalities. First, and most critically, maximal safe resection provides the most immediate and effective decompression of the spinal cord, rapidly halting neurological deterioration and creating the optimal environment for potential recovery. This is paramount in the cervical region, where the risk of catastrophic quadriparesis is high (6, 15, 35). Second, surgery offers a definitive histopathological diagnosis, which is crucial for guiding subsequent systemic therapy, especially in the era of targeted agents and immunotherapy (9). Finally, surgical cytoreduction can enhance the efficacy of adjuvant therapies by reducing tumor burden.

Evidence increasingly supports this aggressive approach. As demonstrated in our case and corroborated by literature, surgery can maintain or improve neurological function and is associated with superior survival outcomes compared to conservative management. For instance, Kalayci et al. (1) reported a mean survival of 9.4 months with surgery vs. 5 months without, while Dam et al. (6) found a median survival of 7.4 months with surgery compared to 2.6 months without. Notably, ISCM from NSCLC appears to be more frequently amenable to surgical resection than from SCLC (10), and satisfactory functional outcomes are achievable (15, 35, 36). In our patient, who underwent C6-T1 laminectomy and microsurgical resection, the advantages of neural tissue decompression, tumor burden reduction, and symptom alleviation were clearly demonstrated, with preserved ambulatory function at follow-up.

From a technical spine surgery perspective, the choice of procedure, such as the laminectomy for posterior access, must be tailored to the lesion. The universal imperative is the utilization of microsurgical techniques to achieve precise dissection at the tumor-spinal cord interface. The use of intraoperative neuromonitoring is strongly advocated to maximize the safety of resection within these eloquent neural tissues.

Patient selection is the keystone of success. The ideal candidate, as exemplified by our 72-year-old patient [who, contrary to some older recommendations (13), benefited significantly despite his age], typically presents with a well-demarcated, solitary metastasis, preserved preoperative neurological function (e.g., ASIA Grade D), and controlled systemic disease. Surgery is also a primary option for radio-resistant tumors or after radiotherapy failure (9, 35). Conversely, it is generally not advised for patients with multisegmental involvement (12), widespread systemic metastases, or a nonfunctional preoperative status (13).

It is important to acknowledge the ongoing debate and the limitations of surgery. Some studies indicate a minimal impact of surgery on postoperative survival (15, 25, 37), highlighting that the decision to operate must be made on a case-by-case basis within a multidisciplinary framework. Furthermore, for large, symptomatic ISCMs carrying a risk of subsequent hemorrhage, microsurgical resection can serve a preventive role, mitigating the risk of rebleeding and sudden neurological deterioration (36).

Lung cancer causes 56% of intramedullary metastases. The median interval from primary diagnosis to metastasis is 6 months. Rising ISCM incidence underscores the importance of early diagnosis/treatment for neurological preservation. Gross total resection may be considered for patients with rapid neurological decline, suitable characteristics, and a well-demarcated focal mass (37). Multimodal therapy improves survival over monotherapy (38). ISCM management depends on primary tumor histology, extracranial spread, treatment strategies, and patient condition. Treatment must be individualized, requiring comprehensive assessment of clinical, radiological, and pathological factors.

### 5.3 Takeaway lessons

This case and literature review offer three critical, actionable lessons for clinicians:

Aggressive Management is Not Contraindicated: An aggressive, multimodal approach should be considered for eligible patients with ISCM. As demonstrated by our case and the literature, a combination of maximal safe resection, modern systemic therapy, and radiotherapy can significantly alter the disease course and preserve neurological function, leading to survival that far exceeds historical expectations.
Implement Vigilant, Long-Term Surveillance: Long-term surveillance is imperative. Our case demonstrates that late local recurrence can occur even after successful initial treatment and can be effectively managed with salvage radiotherapy, emphasizing the need for continuous clinical and radiological follow-up.
Embrace a Personalized, Evolving Paradigm: The treatment paradigm is evolving beyond traditional modalities. Molecular profiling is essential to unlock the potential of targeted therapies and immunotherapy, which are becoming crucial components of successful management. Treatment must be individualized based on a comprehensive assessment of clinical, radiological, and pathological factors.

### 5.4 Patient perspective

The patient was informed about the nature of this case report and the use of his clinical data and images for educational and publication purposes. He provided written informed consent for the publication of this report, expressing hope that his experience might benefit other patients facing a similar diagnosis.

## 6 Conclusion

NSCLC-derived intramedullary spinal cord metastasis (ISCM) represents one of the most formidable challenges in spinal oncology. A high index of suspicion is required, particularly in middle-aged males with cervical cord involvement who present with radicular pain, sensorimotor deficits, and sphincter dysfunction. Acute myelopathy in these patients warrants immediate spinal MRI. This report provides spine surgeons with an evidence-supported, comprehensive, and surgical-led multimodal paradigm. This strategy prioritizes maximal safe resection for well-demarcated lesions to achieve immediate decompression and pathologic diagnosis, effectively integrated with adjuvant radiotherapy and modern systemic therapies. For carefully selected patients, this approach can achieve survival and functional outcomes that far exceed historical expectations. Prospective multicenter studies are warranted to further refine this surgical-led multimodal paradigm, particularly evaluating the role of regimens like nedaplatin in elderly patients.

## Data Availability

The original contributions presented in the study are included in the article/Supplementary Material, further inquiries can be directed to the corresponding author.
