# Supplementary material for: Case Report: Management of cervical intramedullary spinal cord metastasis from NSCLC with a literature review
Source: Front Surg. 2026 Feb 25;13:1760091. doi: 10.3389/fsurg.2026.1760091 (PMC12975956; doi:10.3389/fsurg.2026.1760091)
Supplement: Supplementary file 1 [file Table1.docx]

**Supplementary Table 1. Summary of cases of ISCM caused by NSCLC**

| **Reference** | **Age** | **gender** | **histology** | **symptoms** | **Location** | **Treatment** | **Survival time** | **status** |
| --- | --- | --- | --- | --- | --- | --- | --- | --- |
| Sherbourne DH et al. (1) | 69 | M | NSCLC | W,P,R | C3-C6 | Ra | 13d | deceased |
| Edelson RN et al. (2) | 43 | F | NSCLC | W | T6-T7 | Ra | 1.4m | alive |
| Puljic S et al. (3) | 64 | M | NSCLC | W | T12-L1 | Sx+Ra | NA | NA |
| Hirose et al. (4) | 57 | M | NSCLC | W,P,S,H | T6-T8 | NA | 3m | deceased |
| Hashizume Y et al. (5) | 50 | F | NSCLC | NA | C4-T1 | NA | NA | deceased |
|  | 39 | F | NSCLC | NA | L2 | NA | NA | deceased |
| Costigan DA et al. (6) | 60 | M | NSCLC | W,P,N | C1-C3 | NA | 10d | deceased |
|  | 60 | M | NSCLC | W,P,N | C3,C4,L1-4 |  | 2.5m | deceased |
|  | 63 | M | NSCLC | W,P,N | T6-T9 |  | 3m | deceased |
|  | 63 | F | NSCLC | W,P,N | T |  | NA | deceased |
|  | 66 | M | NSCLC | W,P,N | multiple |  | NA | deceased |
|  | 56 | M | NSCLC | W,P,N | multiple |  | NA | deceased |
|  | 36 | M | NSCLC | W,P,N | T |  | NA | deceased |
| Findlay JM et al. (7) | 74 | M | NSCLC | W,B-S | C1-C3 | Sx+Ra | 3m | alive |
| Tognetti et al. (8) | 59 | M | NSCLC | N,G | C3-C7 | Sx+Ra | 11m | deceased |
|  | 61 | M | NSCLC | W,P,S | T12-L1 | Sx+Ra | 7m | deceased |
| Koelman et al. (9) | 54 | F | NSCLC | W,P | T10-T11 | Sx+Ra | 13m | alive |
| Aoki et al. (10) | 75 | M | NSCLC | W,P,S | L3 | NA | 2w | deceased |
| Jayasundera et al. (11) | 59 | M | NSCLC | P | C2-C3 | Ra | 11m | alive |
| Keung et al. (12) | 31 | F | NSCLC | W,P,N | T4-T5 | Ra | 10w | alive |
| Connolly et al. (13) | 62 | M | NSCLC | W,N | C4-C6 | Sx+Ra+Ch | 11m | deceased |
|  | 35 | F | NSCLC | W,P,B-S | C3-C4 | Sx+Ra+Ch | 4.5m | deceased |
| Mortimer et al. (14) | 69 | M | NSCLC | W,N,S | T12 | Ch+Ra | 2w | NA |
| Wada et al. (15) | 36 | F | NSCLC | W,N,B-S | C3-4,C7-T1 | Ch+Ra | 16m | deceased |
| Komori et al. (16) | 64 | M | NSCLC | W,P | C,T, L | NA | NA | NA |
| Potti et al. (17) | 62 | M | NSCLC | N | C4-C6 | Ch+Ra | 4.5m | deceased |
|  | 52 | M | NSCLC | P | C2-C3 | Ch+Ra | 6.3m | deceased |
|  | 69 | F | NSCLC | W | C4-C5 | Ch+Ra | 1.3m | deceased |
|  | 51 | M | NSCLC | P | C2-C4 | Ch+Ra | 23.5m | deceased |
|  | 54 | M | NSCLC | N | C2-C3 | Ch+Ra | 16.5m | deceased |
|  | 68 | F | NSCLC | N | C4-C6 | Ch+Ra | 9.3m | deceased |
|  | 62 | M | NSCLC | P | C3-C4 | Ch+Ra | 10m | deceased |
| Tashima et al. (18) | 77 | M | NSCLC | W,N,S | L1-L2 | Sx | NA | NA |
| Aryan et al. (19) | 59 | M | NSCLC | B-S | C4-C5 | Sx+Ra+Ch | ＞8m | alive |
| Kalayci et al. (20) | 72 | M | NSCLC | P | C4-C6 | Sx+Ch | ＞8m | alive |
| Watanabe et al. (21) | 53 | M | NSCLC | W,S | T12 | Ra | 12m | deceased |
| Guppy et al. (22) | 54 | M | NSCLC | W,P,N,S | conus | Sx+Ra+Ch | 4m | deceased |
| Marquart et al. (23) | 74 | M | NSCLC | P,N,S | C4-C5 | Sx+Ra | 18w | deceased |
| Conill et al. (24) | 80 | M | NSCLC | P,N | T11-T12 | Ch+Ra | 5m | NA |
|  | 72 | M | NSCLC | S | T12 | Ch+Ra | ＜1m | NA |
|  | 53 | M | NSCLC | W,N | C2-C4 | Ch+Ra | 15m | NA |
| Lee et al. (25) | 32 | M | NSCLC | NA | NA | Ch+Ra | 0.8m | deceased |
|  | 77 | M | NSCLC | NA | NA | Ch+Ra | 1m | deceased |
|  | 73 | M | NSCLC | NA | NA | Refuse | 18.4m | deceased |
| Dam-Hieu et al. (26) | 43 | M | NSCLC | P | C3-C6 | Sx | 2m | deceased |
|  | 68 | M | NSCLC | W,S | T5 | refuse | 4d | deceased |
|  | 47 | F | NSCLC | P,N,S | C2 | Sx+Ra | 8m | deceased |
|  | 66 | M | NSCLC | P,S | conus | refuse | 1m | deceased |
|  | 57 | M | NSCLC | W | conus | Sx+Ra+Ch | 4m | deceased |
|  | 66 | M | NSCLC | W | T9 | PT | 2w | deceased |
|  | 55 | M | NSCLC | W,N,S | conus | Sx+Ra | 11m | deceased |
| Hashii H et al. (27) | 49 | M | NSCLC | NA | NA | Ra | 8w | deceased |
|  | 55 | M | NSCLC |  |  | Ra | 26w | deceased |
|  | 57 | M | NSCLC |  |  | Ra | 9w | deceased |
|  | 64 | F | NSCLC |  |  | Ra | 17w | deceased |
|  | 65 | M | NSCLC |  |  | Ra | 4w | alive |
|  | 76 | M | NSCLC |  |  | Ra | 20w | alive |
| Liu WC et al. (28) | 44 | M | NSCLC | W,P,N | C4 | Sx+Ra+Ch | 8m | deceased |
| Hata et al. (29) | 35 | M | NSCLC | P,N | T7-T8 | Ch+Ra | 7y | alive |
| nadkarni et al. (30) | 46 | M | NSCLC | W,S | conus | Sx+Ra+Ch | 2m | alive |
| Gainor et al. (31) | 31 | M | NSCLC | P,N | C3-C5 | Ra | 5m | NA |
| Mori et al. (32) | 67 | M | NSCLC | G | C1-C2 | Ra | 10m | deceased |
|  | 44 | M | NSCLC | N | C6-C8 | Ra | 22m | deceased |
|  | 55 | M | NSCLC | P | C1-C2 | Ra | 5m | deceased |
| Payer et al. (33) | 77 | M | NSCLC | N | T12-L1 | Sx | 3m | NA |
|  | 27 | M | NSCLC | W,N | T5-T6 | Sx+Ch | 13m | NA |
|  | 60 | M | NSCLC | W,N | C4-C5 | Sx+Ra | 3m | NA |
|  | 54 | M | NSCLC | P,N | T12-L1 | Sx | NA | NA |
| Nayman et al. (34) | 60 | F | NSCLC | P,N | T11-T12 | NA | NA | NA |
| Kumar et al. (35) | 57 | M | NSCLC | W,P,N | T11-T12 | Sx | NA | NA |
| Hommadi et al. (36) | 64 | M | NSCLC | W,S | L1-L2 | Ch+Ra | 6m | deceased |
|  | 62 | M | NSCLC | W,R | C6-C7 | Ra | 4m | deceased |
| Madhavan et al. (37) | 70 | F | NSCLC | N | C2-C3 | NA | NA | NA |
| Kanematsu et al. (38) | 22 | M | NSCLC | W,N | C4-C7 | Sx+Ra+Ch | 14y | alive |
| [Kritikos et al.](https://pubmed.ncbi.nlm.nih.gov/?sort=date&term=Kritikos+M&cauthor_id=38319484) (39) | 68 | M | NSCLC | W | C2-C3 | Sx+Ra+Ch | NA | NA |
| Fujii et al. (40) | 74 | M | NSCLC | W,S,G | C7-T1 | Sx+Ch | 24m+ | alive |
| Current case | 72 | M | NSCLC | W,P,S,G | C7 | Sx+Ra+Ch | ＞18m | alive |
| NSCLC: non-small cell lung carcinoma; W: muscle weakness or paraplegia; P: pain or rachialgia or sciatica; N: numbness or loss of sensation; S: sphincter failure; H: Horner syndrome; G: gait disorders; R: respiratory disease; B-S: Brown-Sequard syndrome; NA: not available; Sx: surgery; Ra: Radiotherapy; Ch: Chemotherapy; PT: Palliative Care; | | | | | | | | |
|  |  |  |  |  |  |  |  |  |

1. Sherbourne DH, Tribe CR, Varma S. INTRAMEDULLARY SPINAL CORD METASTASES: A CLINICO-PATHOLOGICAL REPORT OF THREE CASES. Paraplegia. 1964;2:100-11.

2. Edelson RN, Deck MD, Posner JB. Intramedullary spinal cord metastases. Clinical and radiographic findings in nine cases. Neurology. 1972;22(12):1222-31.

3. Puljic S, Batnitzky S, Yang WC, Schechter MM. Metastases to the medulla of the spinal cord: myelographic features. Radiology. 1975;117(1):89-91.

4. Hirose G, Shimazaki K, Takado M, Kosoegawa H, Ohya N, Mukawa A. Intramedullary spinal cord metastasis associated with pencil-shaped softening of the spinal cord: case report. J Neurosurg. 1980;52(5):718-21.

5. Hashizume Y, Hirano A. Intramedullary spinal cord metastasis. Pathologic findings in five autopsy cases. Acta Neuropathol. 1983;61(3-4):214-8.

6. Costigan DA, Winkelman MD. Intramedullary spinal cord metastasis. A clinicopathological study of 13 cases. J Neurosurg. 1985;62(2):227-33.

7. Findlay JM, Bernstein M, Vanderlinden RG, Resch L. Microsurgical resection of solitary intramedullary spinal cord metastases. Neurosurgery. 1987;21(6):911-5.

8. Tognetti F, Lanzino G, Calbucci F. Metastases of the spinal cord from remote neoplasms. Study of five cases. Surg Neurol. 1988;30(3):220-7.

9. Koelman JH, De Visser M, Kuster JA, Dreissen JJ, Valk J, Koster PA. Intramedullary spinal cord metastasis following a slowly progressive course. J Neurol Neurosurg Psychiatry. 1989;52(12):1451-2.

10. Aoki H, Fujimoto H, Harada K, Tomonari A, Nakamura Y, Kanazawa K, et al. Intramedullary spinal cord metastasis from lung cancer presenting with paraparesis: an autopsied case. Tokushima J Exp Med. 1992;39(1-2):89-93.

11. Jayasundera MV, Thompson JF, Fulham MJ. Intramedullary spinal cord metastasis from carcinoma of the lung: detection by positron emission tomography. Eur J Cancer. 1997;33(3):508-9.

12. Keung YK, Cobos E, Whitehead RP, Roberson GH. Secondary syringomyelia due to intramedullary spinal cord metastasis. Case report and review of literature. Am J Clin Oncol. 1997;20(6):577-9.

13. Connolly ES, Jr., Winfree CJ, McCormick PC, Cruz M, Stein BM. Intramedullary spinal cord metastasis: report of three cases and review of the literature. Surg Neurol. 1996;46(4):329-37; discussion 37-8.

14. Mortimer N, Hughes D, O'Byrne KJ. Intramedullary spinal cord metastasis. Lancet Oncol. 2001;2(10):607.

15. Wada H, Ieki R, Ota T, Iguchi M, Yuasa K, Okamura T, et al. [Intramedullary spinal cord metastasis of lung adenocarcinoma causing Brown-Séquard Syndrome]. Nihon Kokyuki Gakkai Zasshi. 2001;39(8):590-4.

16. Komori T, Delbeke D. Leptomeningeal carcinomatosis and intramedullary spinal cord metastases from lung cancer: detection with FDG positron emission tomography. Clin Nucl Med. 2001;26(11):905-7.

17. Potti A, Abdel-Raheem M, Levitt R, Schell DA, Mehdi SA. Intramedullary spinal cord metastases (ISCM) and non-small cell lung carcinoma (NSCLC): clinical patterns, diagnosis and therapeutic considerations. Lung Cancer. 2001;31(2-3):319-23.

18. Tashima M, Ono N, Noguchi T, Ishikawa H, Kamakari K, Terada Y. [Two cases of intramedullary spinal cord metastasis of lung cancer detected with MRI]. Nihon Kokyuki Gakkai Zasshi. 2003;41(4):320-3.

19. Aryan HE, Farin A, Nakaji P, Imbesi SG, Abshire BB. Intramedullary spinal cord metastasis of lung adenocarcinoma presenting as Brown-Sequard syndrome. Surg Neurol. 2004;61(1):72-6.

20. Kalayci M, Cağavi F, Gül S, Yenidünya S, Açikgöz B. Intramedullary spinal cord metastases: diagnosis and treatment - an illustrated review. Acta Neurochir (Wien). 2004;146(12):1347-54; discussion 54.

21. Watanabe M, Nomura T, Toh E, Sato M, Mochida J. Intramedullary spinal cord metastasis: a clinical and imaging study of seven patients. J Spinal Disord Tech. 2006;19(1):43-7.

22. Guppy KH, Wagner F. Metastasis to the conus medullaris: case report. Neurosurgery. 2006;59(5):E1148; discussion E.

23. Marquart C, Weckesser M, Schueller P, Hasselblatt M, Wassmann H, Schröder J. Intramedullary spinal cord metastasis as initial presentation of systemic cancer--report of a rare case. Zentralbl Neurochir. 2007;68(4):214-6.

24. Conill C, Marruecos J, Verger E, Berenguer J, Lomeña F, Domingo-Domènech J, et al. Clinical outcome in patients with intramedullary spinal cord metastases from lung cancer. Clin Transl Oncol. 2007;9(3):172-6.

25. Lee SS, Kim MK, Sym SJ, Kim SW, Kim WK, Kim SB, et al. Intramedullary spinal cord metastases: a single-institution experience. J Neurooncol. 2007;84(1):85-9.

26. Dam-Hieu P, Seizeur R, Mineo JF, Metges JP, Meriot P, Simon H. Retrospective study of 19 patients with intramedullary spinal cord metastasis. Clin Neurol Neurosurg. 2009;111(1):10-7.

27. Hashii H, Mizumoto M, Kanemoto A, Harada H, Asakura H, Hashimoto T, et al. Radiotherapy for patients with symptomatic intramedullary spinal cord metastasis. J Radiat Res. 2011;52(5):641-5.

28. Liu WC, Chung CL, Chai CY, Tan LB, Wang CJ, Kwan AL. Metachronous brain and intramedullary spinal cord metastases from nonsmall-cell lung cancer: a case report. Kaohsiung J Med Sci. 2012;28(5):289-93.

29. Hata Y, Takai Y, Takahashi H, Takagi K, Isobe K, Hasegawa C, et al. Complete response of 7 years' duration after chemoradiotherapy followed by gefitinib in a patient with intramedullary spinal cord metastasis from lung adenocarcinoma. J Thorac Dis. 2013;5(2):E65-7.

30. Mavani SB, Nadkarni TD, Goel NA. Intramedullary conus metastasis from carcinoma lung. J Craniovertebr Junction Spine. 2013;4(1):40-2.

31. Gainor JF, Ou SH, Logan J, Borges LF, Shaw AT. The central nervous system as a sanctuary site in ALK-positive non-small-cell lung cancer. J Thorac Oncol. 2013;8(12):1570-3.

32. Mori Y, Hashizume C, Shibamoto Y, Kobayashi T, Nakazawa H, Hagiwara M, et al. Stereotactic radiotherapy for spinal intradural metastases developing within or adjacent to the previous irradiation field--report of three cases. Nagoya J Med Sci. 2013;75(3-4):263-71.

33. Payer S, Mende KC, Westphal M, Eicker SO. Intramedullary spinal cord metastases: an increasingly common diagnosis. Neurosurg Focus. 2015;39(2):E15.

34. Nayman A, Özbek S, Temizöz O, Kanat F, Kıvrak AS. Spinal intramedullary metastasis as the first manifestation of lung cancer. Spine J. 2015;15(10):e9-10.

35. Kumar JI, Yanamadala V, Shin JH. Intramedullary spinal metastasis of a carcinoid tumor. J Clin Neurosci. 2015;22(12):1990-1.

36. Hommadi M, Belemlih M, Marnouch E, Maghous A, Zaghba N, Hamidi FZ, et al. Intramedullary spinal cord metastases: Report of three cases and review of the literature. Cancer Radiother. 2021;25(2):169-74.

37. Madhavan AA, Diehn FE, Rykken JB, Wald JT, Wood CP, Schwartz KM, et al. The Central Dot Sign : A Specific Post-gadolinium Enhancement Feature of Intramedullary Spinal Cord Metastases. Clin Neuroradiol. 2021;31(2):383-90.

38. Kanematsu R, Hanakita J, Takahashi T, Minami M, Mitsuya K. Long-term survival following molecular-targeted therapy for intramedullary non-small-cell lung cancer metastasis. Surg Neurol Int. 2024;15:312.

39. Kritikos M, Vivanco-Suarez J, Teferi N, Lee S, Kato K, Eschbacher KL, et al. Survival and neurological outcomes following management of intramedullary spinal metastasis patients: a case series with comprehensive review of the literature. Neurosurg Rev. 2024;47(1):75.

40. Fujii R, Morozumi M, Muramoto A, Matsubara Y. A case of sustained neurological improvement in a metastatic intramedullary spinal cord tumor from lung cancer treated with immune checkpoint inhibitor therapy. Eur Spine J. 2025;34(8):3474-80.
